# A plan template‐based automation solution using a commercial treatment planning system

**DOI:** 10.1002/acm2.12848

**Published:** 2020-03-16

**Authors:** Xiaotian Huang, Hong Quan, Bo Zhao, Wing Zhou, Charles Chen, Yan Chen

**Affiliations:** ^1^ School of Physics and Technology Wuhan University Wuhan China; ^2^ Elekta (Shanghai) Instruments Ltd Shanghai China; ^3^ Department of Radiation Oncology Peking University First Hospital Beijing China

**Keywords:** auto‐planning, inverse planning, template, VMAT

## Abstract

**Purpose:**

The purpose of this study was to develop an auto‐planning platform to be interfaced with a commercial treatment planning system (TPS). The main goal was to obtain robust and high‐quality plans for different anatomic sites and various dosimetric requirements.

**Methods:**

Monaco (Elekta, St. Louis, US) was the TPS in this work. All input parameters for inverse planning could be defined in a plan template inside Monaco. A software tool called Robot Framework was used to launch auto‐planning trials with updated plan templates. The template modifier external to Monaco was the major component of our auto‐planning platform. For current implementation, it was a rule‐based system that mimics the trial‐and‐error process of an experienced planner. A template was automatically updated by changing the optimization constraints based on dosimetric evaluation of the plan obtained in the previous trial, along with the data of the iterative optimization extracted from Monaco. Treatment plans generated by Monaco with all plan evaluation criteria satisfied were considered acceptable, and such plans would be saved for further evaluation by clinicians. The auto‐planning platform was validated for 10 prostate and 10 head‐and‐neck cases in comparison with clinical plans generated by experienced planners.

**Results:**

The performance and robustness of our auto‐planning platform was tested with clinical cases of prostate and head and neck treatment. For prostate cases, automatically generated plans had very similar plan quality with the clinical plans, and the bladder volume receiving 62.5 Gy, 50 Gy, and 40 Gy in auto‐plans was reduced by 1%, 3%, and 5%, respectively. For head and neck cases, auto‐plans had better conformity with reduced dose to the normal structures but slightly higher dose inhomogeneity in the target volume. Remarkably, the maximum dose in the spinal cord and brain stem was reduced by more than 3.5 Gy in auto‐plans. Fluence map optimization only with less than 30 trials was adequate to generate acceptable plans, and subsequent optimization for final plans was completed by Monaco without further intervention. The plan quality was weakly dependent on the parameter selection in the initial template and the choices of the step sizes for changing the constraint values.

**Conclusion:**

An automated planning platform to interface with Monaco was developed, and our reported tests showed preliminary results for prostate and head and neck cases.

## 1 Introduction

Due to the increasing number of patients, automated treatment planning is highly demanded in radiation oncology. A variety of knowledge‐based and learning‐based auto‐planning methods have been explored in order to improve both the workflow efficiency and plan quality consistency. Scripting‐based auto‐planning has been commercially available on several treatment planning systems (TPSs). Current methods were primarily focused on how to control the scripts, possibly by guidance with predicted rewards for achievable dosimetric goals based on a prior knowledge. Wang^1^, ^2^ proposed an autopilot scheme via scripting by recording the interactions between planners and TPS, such as the step sizes for adjusting dosimetric parameters in each optimization. Such an approach allowed the system to mimic the process of planning by a skilled planner. A potential drawback was that most adjustment steps were fixed. A more flexible method was introduced by Yan^3^, ^4^ using an AI‐guided coordination for parameter adjustments of various variables, and clinical application of this fuzzy inference system (FIS) was implemented for site‐specific cases.^5^ Furthermore, a further adaptive neuro fuzzy inference system (ANFIS) was advanced by Stieler,^6^ which generated an increased learning capability over FIS for complicated planning cases with more efficient use of the prior data. Breedveld^7^, ^8^ developed a lexicographic multicriteria optimization method called Erasmus‐iCycle for deriving a Pareto optimal solution in an acceptable time and connected this with Monaco. A distinguished feature of Erasmus‐iCycle was its beam angle optimization for IMRT. The Erasmus‐iCycle planning was validated for the treatment of spinal metastases by VMAT^9^ and for prostate cancers.^10^ This method required a wish‐list creation with treatment site‐specific clinical data. Model generalization could be a difficulty for large‐scale clinical application since less organized data of physician preferred treatment protocol should be required. A powerful database seemed necessary for clinical implementation of the technology. As in Fan,^11^ the kernel density estimation method^12^ was used in combination with optimized training datasets for auto‐planning breast and rectal cancer cases.

To set up appropriate objectives and constraints for specified plan acceptance criteria is the key to achieve an acceptable result in inverse planning. There are two main problems for auto‐planning with a validated TPS that does not provide a complete auto‐planning solution. One is to automatically drive the TPS to work through its internal optimization process with a selected set of objectives and constraints, and the other is to optimize the parameters of these objectives and constraints for initializing the TPS automatically by repeated planning trials. With Monaco, the first problem can be solved using a plan template, which selects the optimization conditions such as the beam arrangement and the cost functions used. The second problem is to search for an optimal set of parameters for Monaco optimization. A clinically acceptable plan can be generated automatically with the optimal parameter set.

Monaco optimization is performed in two stages,^13^ the fluence map optimization (FMO) in Stage I, and MLC segmentation followed with segment shape and segment weight optimization in Stage II. As a practical matter, auto‐planning only needs to obtain an acceptable FMO plan. The Stage II optimization is performed only once for the interest of efficiency.

This work aimed at improving the efficiency and robustness of auto‐planning of IMRT. The automation process used a clinically validated TPS and it was driven by a plan template and scripting, similar to previous techniques like the AutoPlan^14^ in Pinnacle (Philips Medical System) and Erasmus‐iCycle. An auto‐planning platform was developed that included a rule‐based template modification module to allow a quick adaptation to different clinical sites and planning protocols through an application program interface (API). The rule‐based program applied a set of general strategies, mimicking the workflow by an experienced planner. For cases with a new planning protocol, the system could automatically generate an initial plan template based on a clinically accepted Monaco plan complied with the planning protocol. The “model” case could be the work by an experienced planner, only it was required to use a minimal set of constraint functions and parameters. Essentially the initial template defined the space of optimization, for example, certain type of cost functions and geometric parameters involved. Subsequently, the plan template was automatically updated based on a systematic plan evaluation along with a standard procedure for changing the constraint parameters. The repeated trials were performed until the result could meet all acceptance criteria.

## 2 Materials and Methods

### 2.1 Retrospective Planning study

In this study, 10 prostate cases and 10 head and neck cases treated with simultaneous‐integrated boost (SIB) were selected for a feasibility test for our auto‐planning system. The patients were treated at Peking University First Hospital using Volumetric Modulated Arc Therapy (VMAT) from June 2016 to December 2017. Clinical plans created by two dosimetrists with Monaco v5.11 were used for comparison. The detailed dose prescription for the target volumes and critical structures of the two case groups were listed in Tables S1 and S2, respectively, in the supplementary materials. For a valid comparison, the automatically generated plans were scaled to cover 95% of the PTV volume by the prescription dose, which was the accepted dose level for the clinical plans. The maximum number of segments, minimum segment size, and minimum MU in MLC sequencing for auto‐planning were identical as used for the clinical plans. The clinical plans and automatically generated plans were compared by a two‐sided Wilcoxon signed‐rank test to assess the statistical significance (*P* < 0.05).

### 2.2 Sensitivity Analysis

**FIG. 1:**
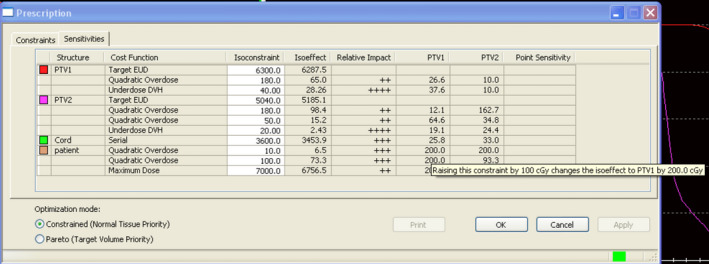
The sensitivity window in Monaco. (Conflicts between constraints and goals).

In IMRT optimization, to find how various constraints should be adjusted to meet all dose constraints simultaneously could be a very cumbersome and time‐consuming procedure. A helpful tool for avoiding fruitless trials is the sensitivity analysis, since it shows the essential conflicts and trade‐offs between goals and constraints. The sensitivity analysis in Monaco^15^ is based on the values of the Lagrange multipliers *λ_i_*, as in Eq. (1), which is the optimality condition for the formal constrained optimization problem

(1) $$\nabla_{\varphi }f^{*}=-\sum_{i=1}^{m}\lambda_{i}^{*}\nabla_{\varphi }g_{{}_{i}},$$

where *f* and *g* are the objective and constraint functions with respect to dose D(φ) and beamlet weight *φ.* The differentials of the objective with respect to the changes of the constraint functions estimate the impact of the constraints to the target dose coverage. The details of relevant argument can be found in Alber et al.^16^ The information is provided in Monaco along with the specified value (Isoconstraint) and current objective value (Isoeffect) of each constraint function, as shown in Fig. 1. For facilitating repeated optimization trials automatically, the objectives and constraints can be adjusted based on dosimetric evaluation of the current plan. This is combined with the trajectories of the Isoconstraints, Isoeffects, and the sensitivities with the weighting adjustment by Monaco for the current plan. In particular, the determination of proper step sizes for changing the parameters of the constraint functions is a critical component for auto‐planning, since the TPS only adaptively changes the relative weights of constraint functions.

### 2.3 Auto‐planning platform

**FIG. 2:**
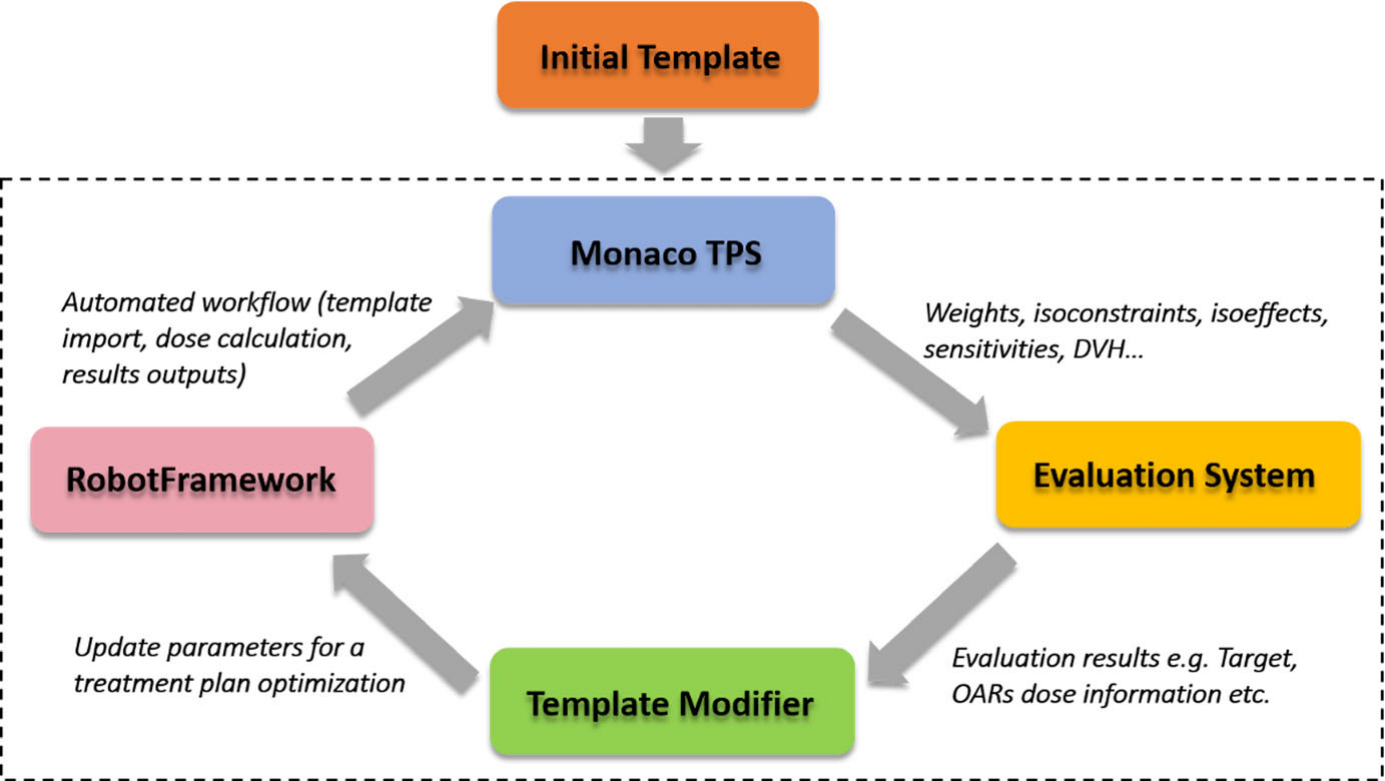
The auto‐planning platform.

The auto‐planning platform consisted of four main components that were interfaced with the Monaco TPS, as seen in the flowchart in Fig. 2. Repeated planning trials were performed so the results could be evaluated improved, just as for a human planner to carry out the process of planning. The major components of our auto‐planning platform directly interacted with TPS. This made it possible to exercise the same strategies of an experienced planner through template modification with a Python program. Each component is introduced as the following.

#### 2.3.1 Robot Framework

**FIG. 3:**
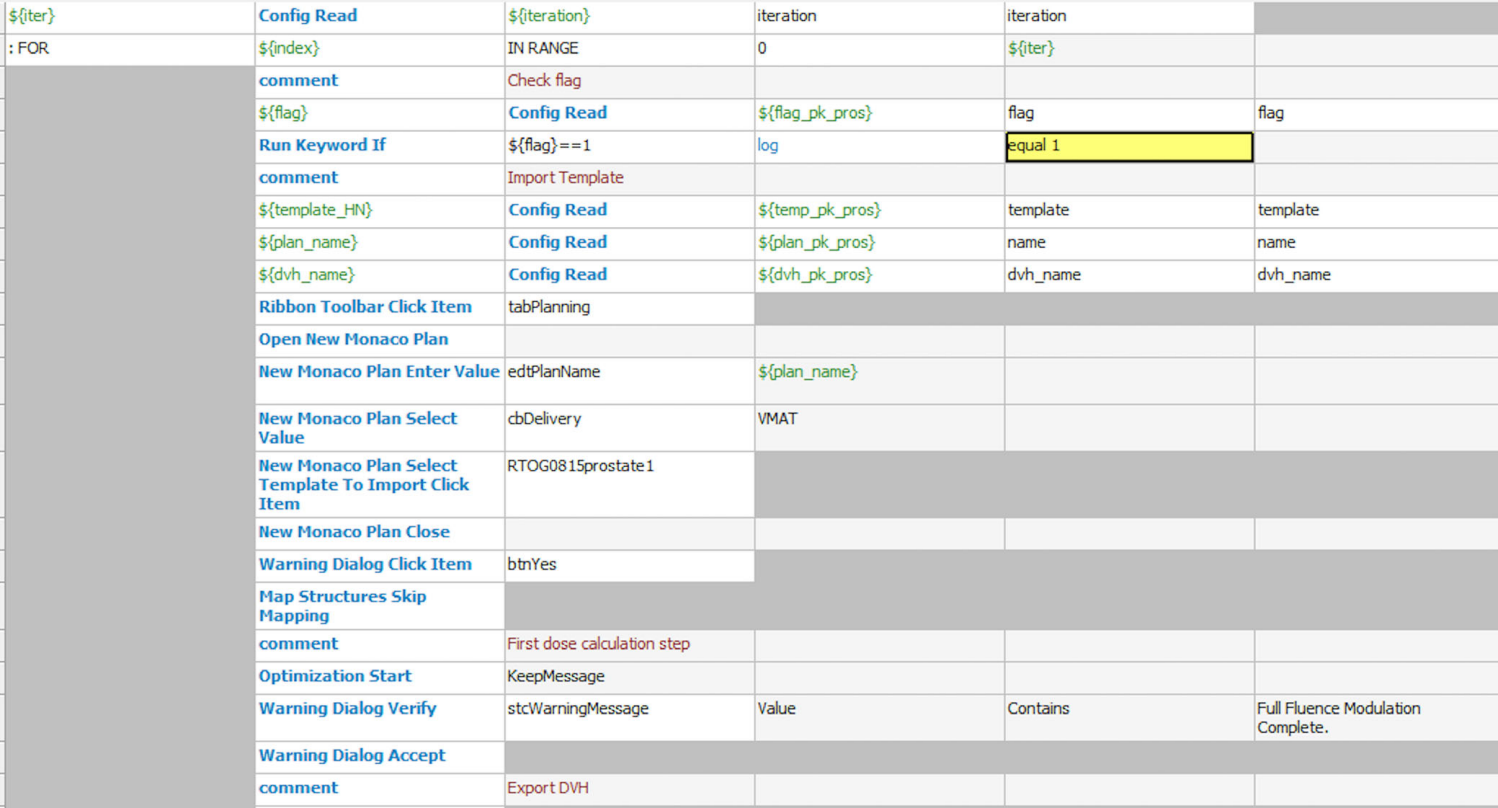
Capture of keywords in Robot Framework.

The Robot Framework (RIDE) was used to automatically operate the TPS. It was similar to a scripting program that enabled three functions for this work: (a) to automatically load a new plan template; (b) to automatically launch the optimization process; (c) to automatically export the dose–volume histogram (DVH) results. RIDE could be seen as a toolbox outside the Monaco TPS. Manual operation with the TPS, such as initializing a new plan, and importing or exporting planning parameters for decisions with updating the IMRT constraints for that next planning trials were replaced by a robot. Each step of the workflow of a planning trial performed with a mouse click by a planner was written as a batch of keywords in RIDE, as illustrated by the screen capture in Fig. 3.

#### 2.3.2 Evaluation System

**FIG. 4:**
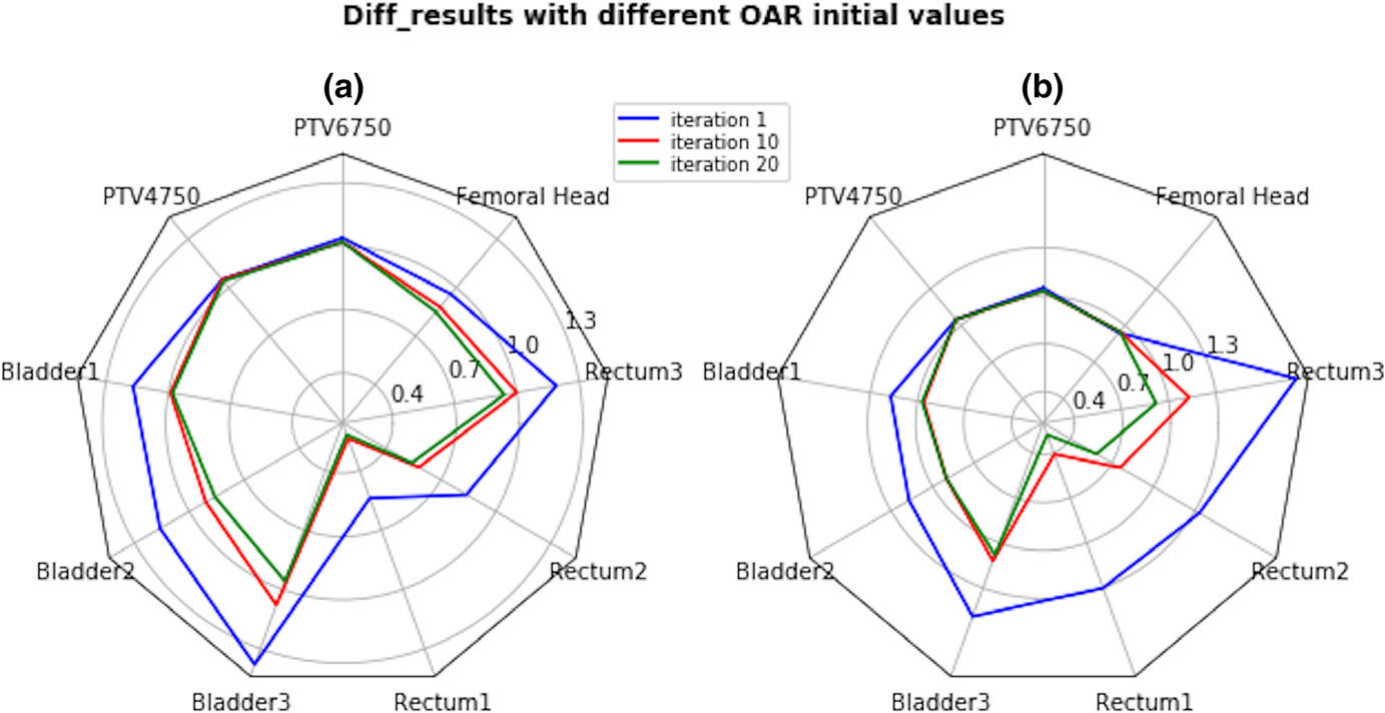
Spider Plots (a) and (b) of three planning iterations starting from different initial constraints on the OARs. If all spokes of a plot (of the same color) are inside the unit circle, the plan is considered acceptable.

For inverse planning, a dosimetric goal on a target volume or OAR was termed as a prescription item. A prescription item could be the maximum, or minimum dose on a volume, or a dose–volume point. How a specific prescription item was achieved in a plan was parameterized by an index, noted as “Diff_result”. For example, for at least 95% of PTV volume to receive the prescribed dose, the Diff_result was defined as the ratio of the achieved percentage volume covered by the prescribed dose and 95%. For OARs, a “Diff_result” value equals to or less than *1* means that the corresponded goal was fully met. Overall the prescription items could then be evaluated with a spider chart,^17^ as shown in Fig. 4. The primary focus in our automated planning was to meet every prescription item or drive its “Diff_result” index close to 1 as much as possible, rather than using a numerical score for ranking the plans. A ranking system could be strongly dependent on physicians’ preference and specific patient anatomy, especially the overlapping and distance between the target volume and an adjacent OARs.^18^ Therefore, a plan would be considered “acceptable” by the evaluation system if every prescription item was met.

#### 2.3.3 Template Modifier

**FIG. 5:**
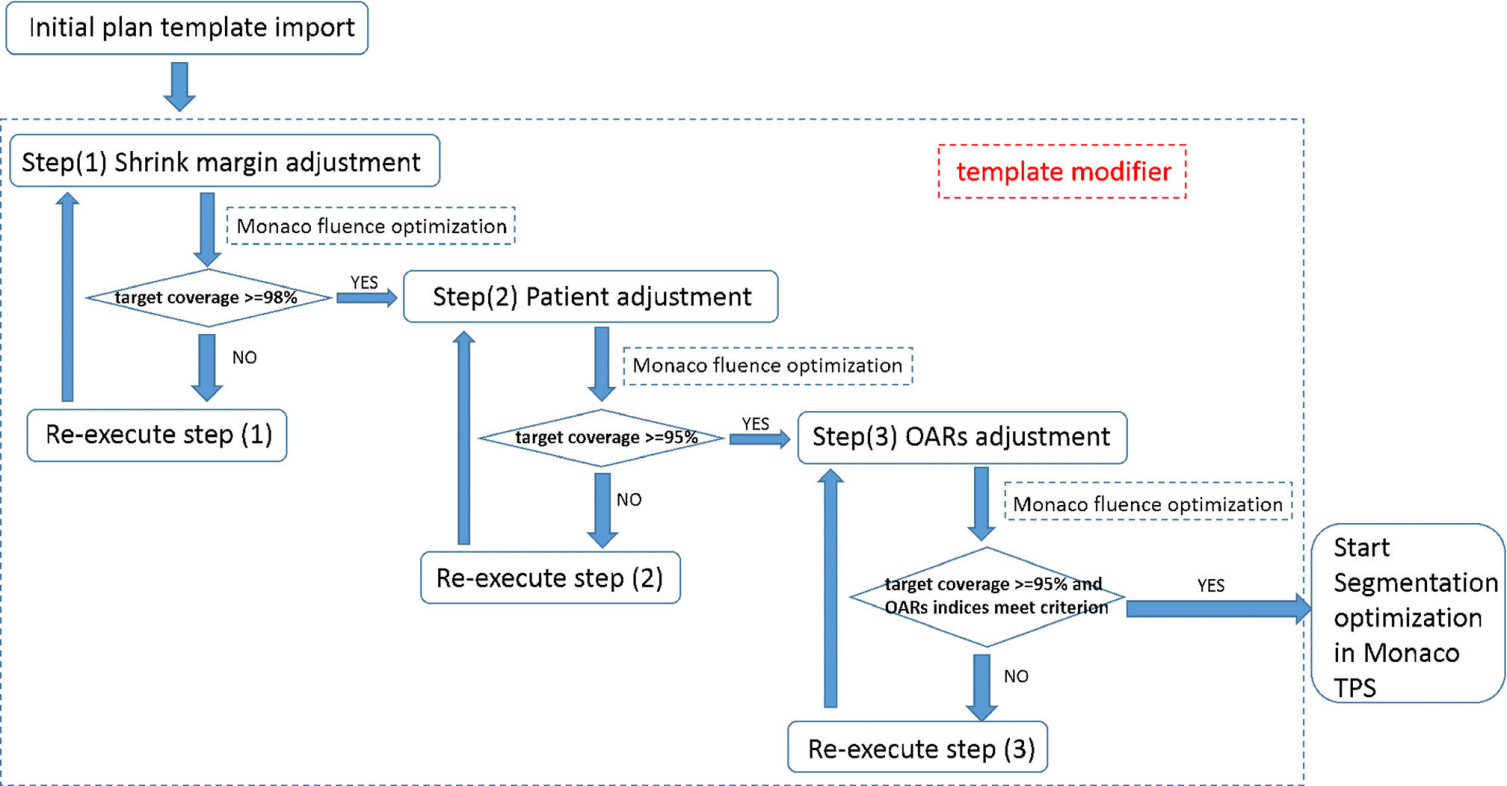
Display of the detailed flowcharts in the template modifier.

The template modifier updated the IMRT parameters in a new template for the next planning trial. The decisions were based on the plan evaluation results and sensitivity analysis data from TPS for the current plan. It was developed with Python (Python 3.6) following a “divide‐and‐conquer” strategy, as often employed by experienced Monaco users. The objective and constraint functions were imposed and modified in three steps, as shown in Fig. 5. Constrained optimization mode was selected in Monaco TPS.

The first step was only for the cases with multiple target volumes and different prescribed dose levels. The purpose was to achieve an adequate dose coverage to the primary target and to satisfy the required dose conformity and uniformity for all targets. Monaco uses a geometric parameter called the shrink margin that allows for reduction of the volume on which the associated constraint function is applied. The shrink margins can control the dose fall‐off where a target overlaps with another structure, either a secondary target with lower prescribed dose or an OAR. Optimal shrink margins for individual constraint functions were determined by improving the conformity in the secondary targets as much as possible while keeping the coverage and required uniformity in the primary target. When Monaco indicated that an overdose constraint had a high impact on the primary target, the constraint would be relaxed by increasing the shrink margins accordingly.

The second step worked in the same way as the first step, but the purpose was to determine optimal constraints on the unspecified normal tissue, often named “patient” or “body” in Monaco. A global maximum dose was often used to impose a hard constraint, and three or four quadratic overdose cost functions (CF) were used to control the 90%, 80%, and 70% isodose surfaces in unspecified normal tissue, respectively. In subsequent trials, the CFs were only adjusted with their specified parameters, defined as the Root Mean Square (RMS) over certain dose level. The dose levels and the shrink margins of the CFs on unspecified normal tissue were determined such that Monaco optimization would see a moderate impact on target coverage. This would leave a space for reshaping the isodose surfaces in the next step with the constraints on each OAR imposed.

**FIG. 6:**
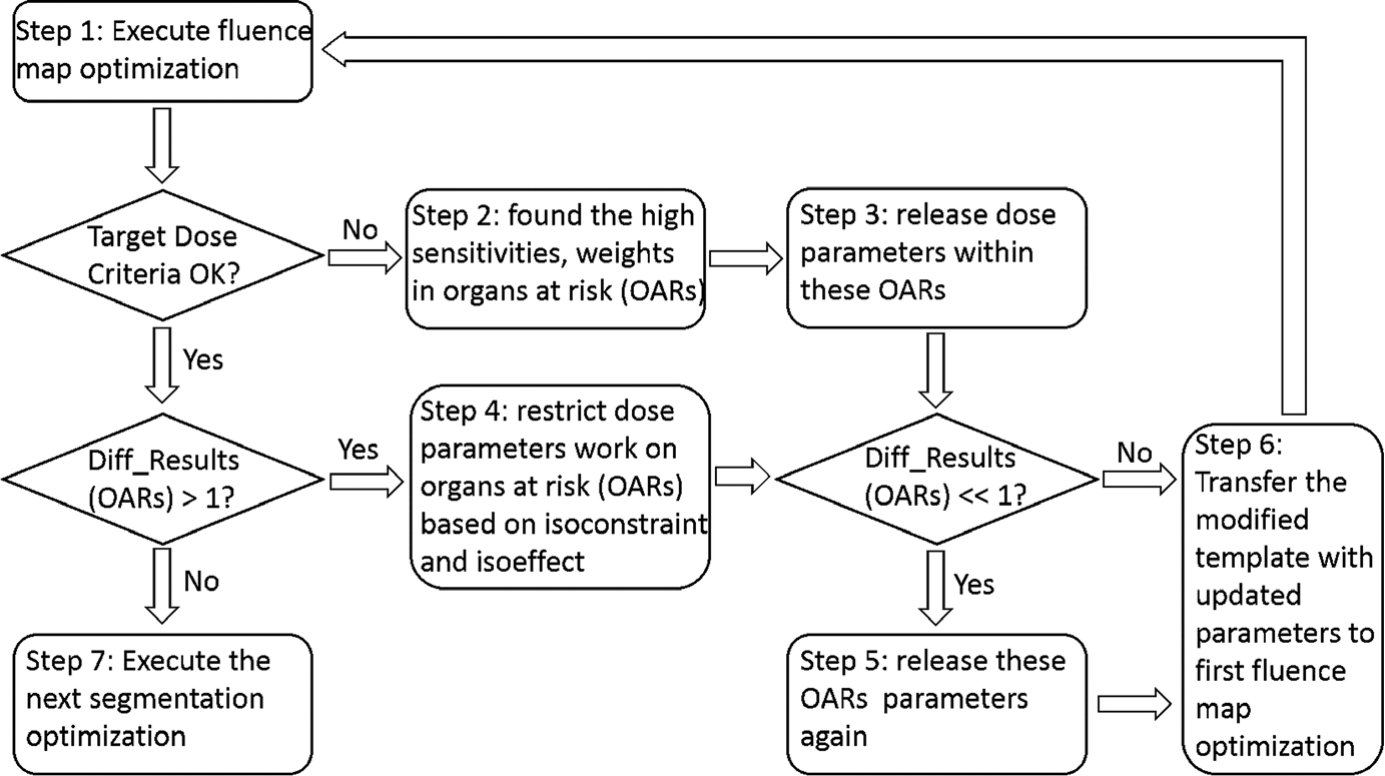
Illustration of details in the third step (OARs adjustment).

In the last step, all the constraints came into effect. The OAR constraints were adjusted adaptively to satisfy the OAR prescription items without losing the target dose coverage. Multivariate dosimetric evaluation and sensitivity analysis were performed to determine how constraint parameters should be updated in mitigating the various conflicts. The details of the process are illustrated in Fig. 6.

The maximum numbers of iterations for the three steps in Template Modifier were set to 15, 15, and 30, respectively. The process was limited to predefined maximum number of trials to avoid a dead loop of fruitless trials.

#### 2.3.4 Initial Template

**FIG. 7:**
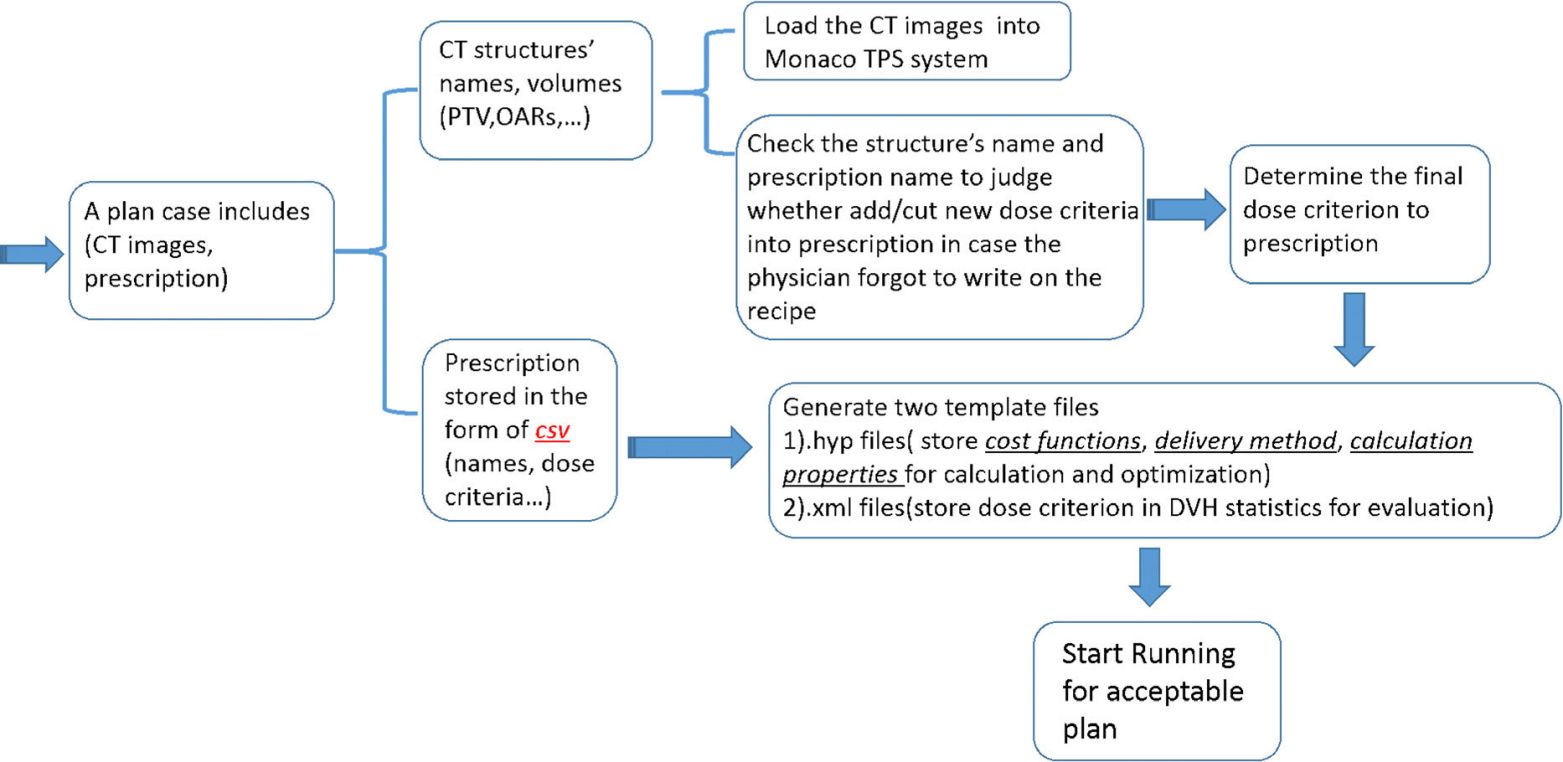
Demonstration of the initial plan template file editor‐based prior information.

A preliminary initial template could be obtained from an example Monaco plan for a similar case to obtain the basic setup information such as delivery technique, dose calculation and MLC segmentation settings, prescribed dose and fraction size, and constraint functions. An initial template editor developed with Python was used to start a new case. The first step was to check the names of the structures defined in the new case and edit the structure names in the template. The second step was to check each prescription item and augment the constraint function set, in order to facilitate auto‐planning with staged selection and setting for the optimization parameters. This would also allow the flexibility of using different cost functions available in Monaco that may affect the detailed dose distribution features, and the plan acceptance criteria for evaluation to be consistent with the prescription for the new case. The template editor is illustrated in Fig. 7.

### 2.4 Treatment planning performance

**FIG. 8:**
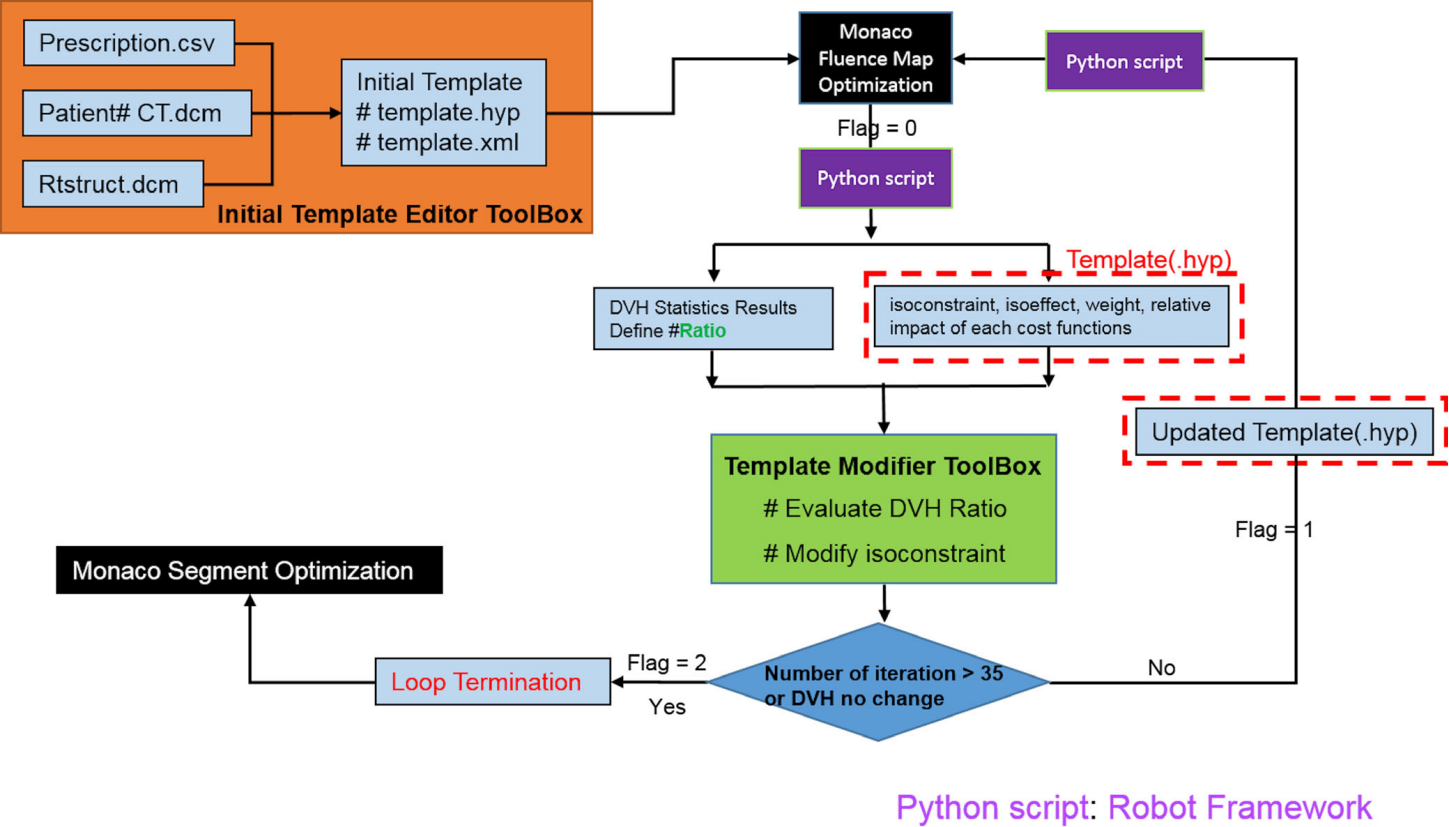
The whole flowchart of auto‐planning platform.

The flowchart of auto‐planning platform is shown in Fig. 8. The main steps performed included (a) plan initialization by a template; (b) dose calculation and fluence optimization; (c) export the plan data, including DVH evaluation, optimization parameters such as Isoconstraints, Isoeffects, Monaco determined weights, and sensitivity for evaluation; (d) updating parameters in the template for the next trial.

## 3 Results

Our tests were performed using a Monaco workstation equipped with two Intel(R) Xeon(R) 2.5 GHz processors (48 cores) and 48 GB RAM. The average optimization time was 30 min for prostate cases, and 50 min for head and neck cases.

Most cases did not need manual interruption as once the initial template was loaded into TPS, all the following steps were automatically finished. Certain cases may need manual interruption if the auto‐plan quality was not acceptable.

### 3.1 Prostate cases

**FIG. 9:**
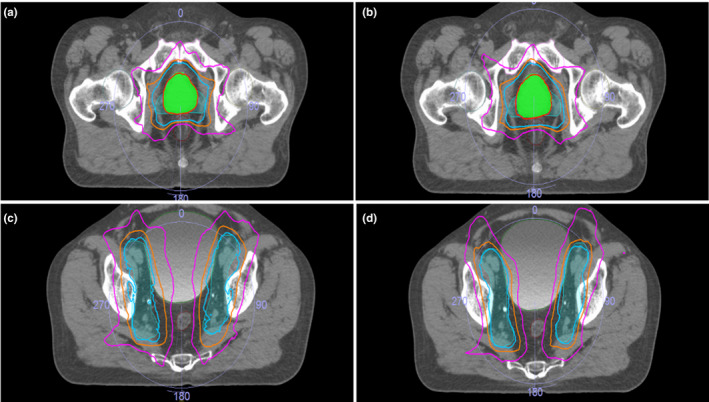
Dose distribution comparison of auto (b, d) and clinical (a, c) prostate planning (red line: 67.5 Gy isodose line; cyan line: 45 Gy isodose line; orange line: 40 Gy isodose line).

**FIG. 10:**
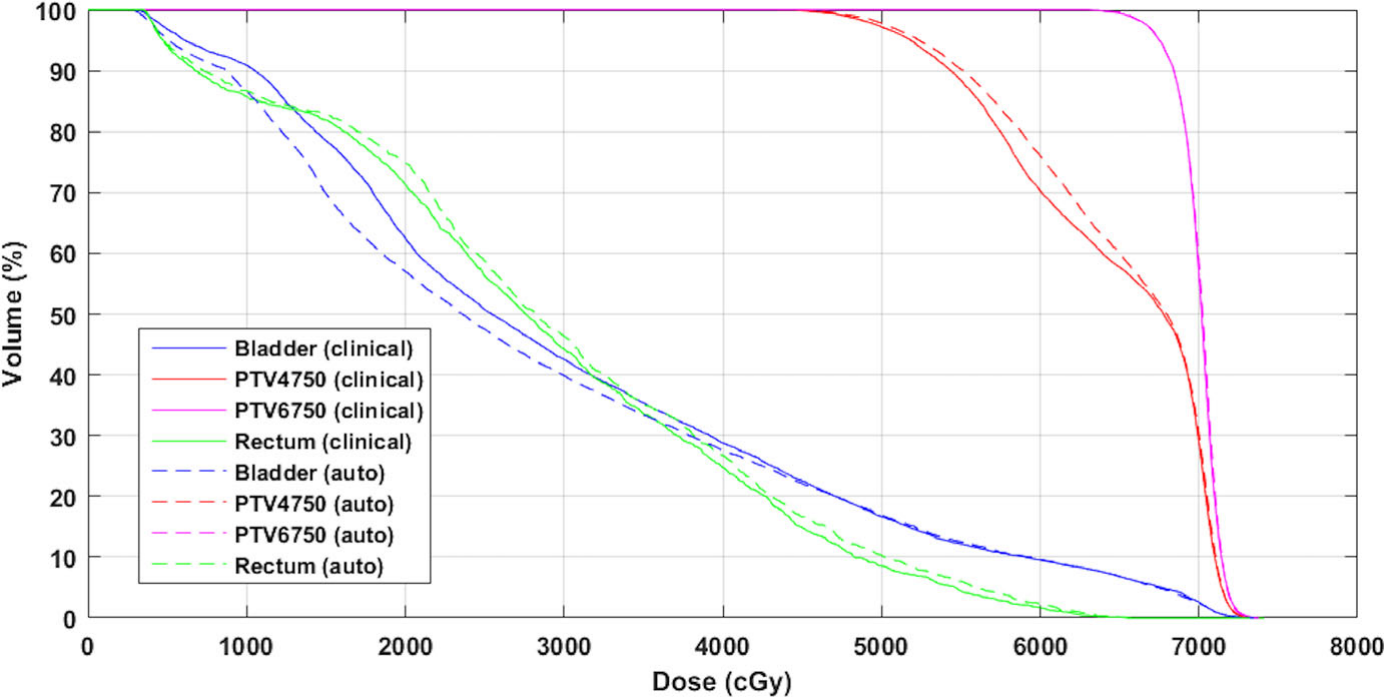
Final DVH comparison between auto and clinical prostate plan. DVH, dose–volume histogram.

The dose distributions of an automatically generated plan and the clinical plan were very similar, as shown by an example in Fig. 9. Better dose conformity and thus better OAR sparing were consistently achieved with the automatically generated plans. The area within the pink color isodose line was smaller. The final plans that were judged acceptable had almost identical target dose coverage as the clinical plans, as seen in the DVH comparison in Fig. 10. The plan evaluation statistical analysis was presented in Table S3 in supplementary materials. The auto‐planning achieved a slight improvement for the bladder, even it started with relatively weaker constraints than that for the PTV and the rectum.

In some cases, an acceptable FMO could be reached in just two trials, while in other cases, more iterations were needed to find an acceptable solution, typically when there was significant overlapping of the PTV and the critical structures.

### 3.2 Head and neck cases

**FIG. 11:**
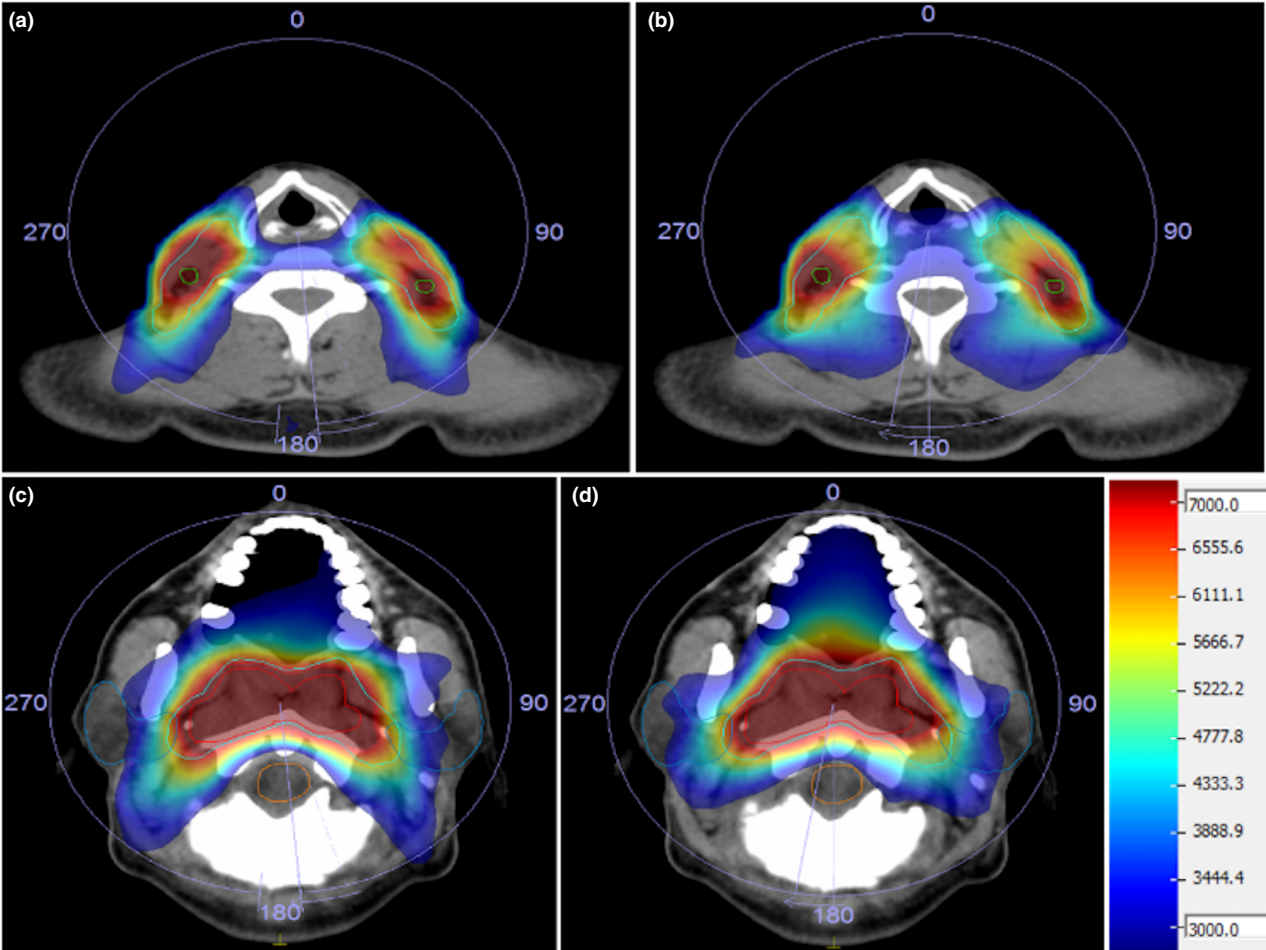
Comparison of the isodose distribution of auto (a, c) and clinical (b, d) plans.

**FIG. 12:**
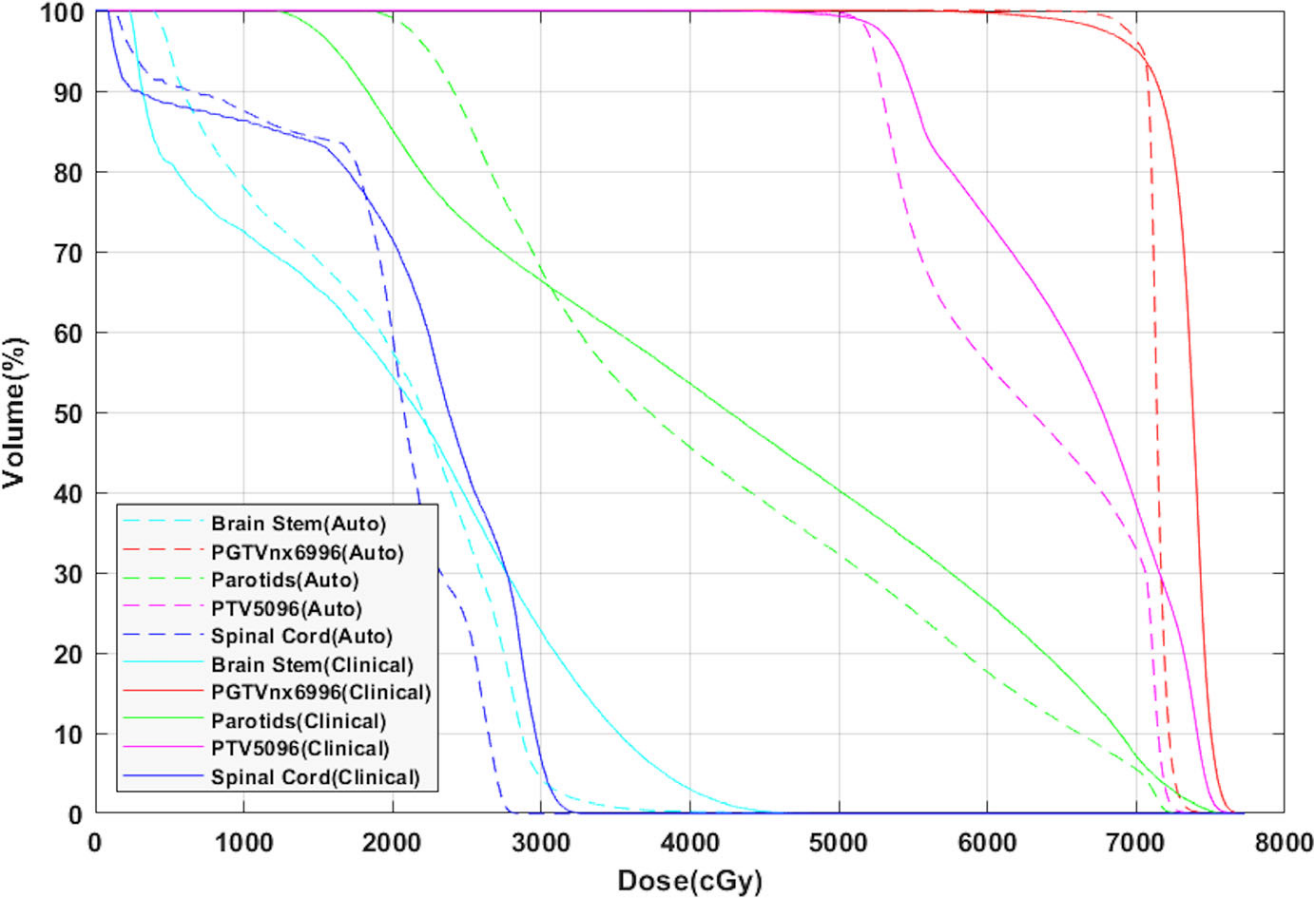
Final DVH comparison between auto and clinical head and neck plans. DVH, dose–volume histogram.

A side‐by‐side comparison of an automatically generated plan and the clinical plan is shown in Fig. 11. The automatically generated plans were clinically acceptable with better OAR sparing, especially for the parotid glands and the brain stem. The dose conformity was better, but the target dose maximum was slightly higher. This was because the system was required to meet all prescription requirements on the OARs. As seen in Fig. 12, parotid and cord dose were significantly improved with a less conformal dose to PTV5096, but PTV5096 conformity was not a prescribed item. The detailed statistics information is listed in Table S4.

For a complicate case like head and neck treatment, auto‐planning needed 20–30 trials to obtain an acceptable plan, including several attempts to choose different combination of cost functions in the initial template. A layer of optimization for the constraint function set, and the presets of certain parameters, was performed. Template optimization was conveniently done with our initial template editor, along with the functionality in Monaco that allowed turning on and off optimization conditions. It did not need to treat all parameter as optimization variables, which would increase the calculation time tremendously. Note that a single optimized initial template was used for all 10 cases tested.

Our results showed that auto‐planning with FMO was adequate to generate acceptable plans. The segment shape and segment weight optimization in Stage II with Monte Carlo dose calculation could reproduce similar dose statistics and distribution based on beamlet approximation. A noticeable difference between the final plan and the FMO plan was an increased maximum dose and a small loss in the target dose coverage. The magnitudes of the differences for prostate cases were less than in the head and neck cases, respectively, indicating that a deteriorate plan quality in Stage II optimization was associated with the complexity of the plan. The detailed statistical comparison between fluence map optimization and segment optimization was listed in Tables S5 and S6 in supplementary materials.

## 4 Discussion

In this work, we developed a new auto‐planning platform for Monaco TPS. The platform included an initial template editor, a template modifier, and a plan quality evaluation system. The template modifier was a rule‐based system in current implementation. Clinical implementation of our auto‐planning platform has the potential to improve both the efficiency and the consistency of the plan quality.

**FIG. 13:**
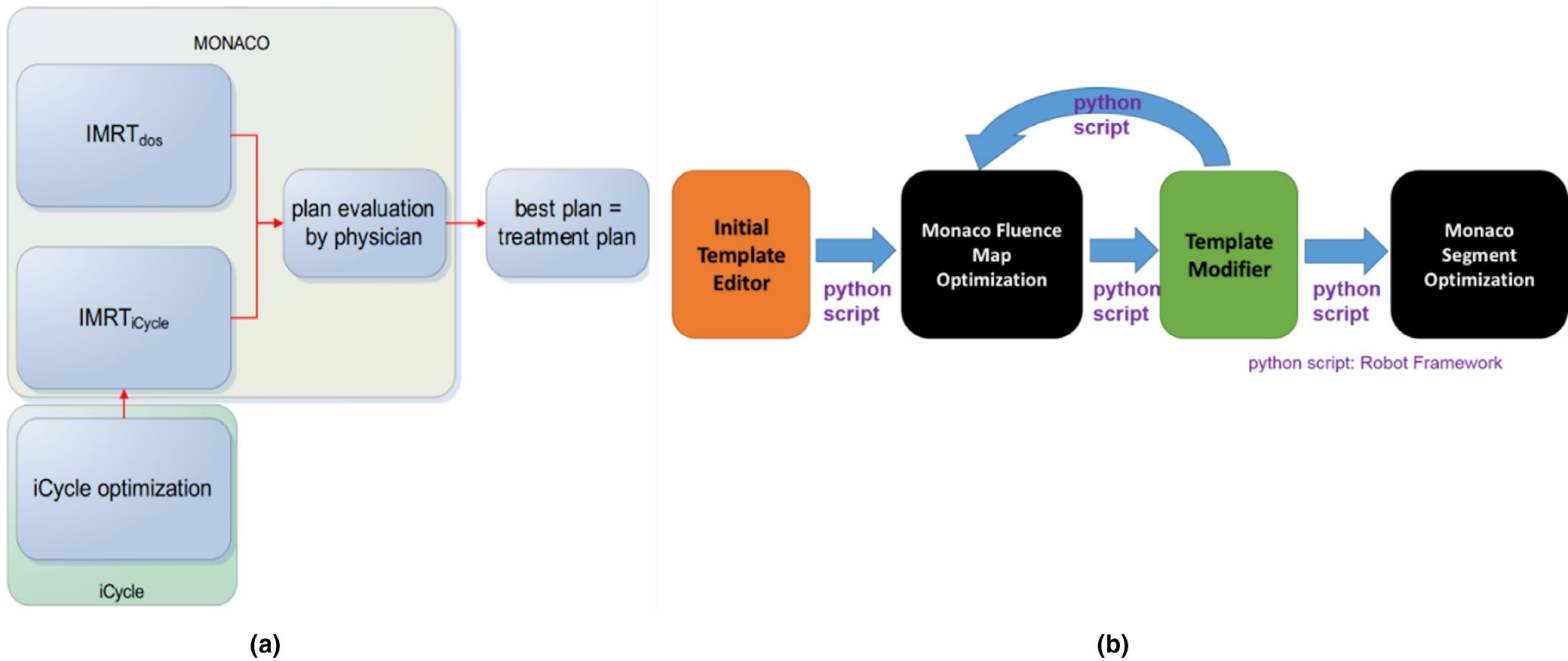
(a) The flowchart of Erasmus‐iCycle; (b) the flowchart of our auto‐planning.

Currently, three different paradigms are used for auto‐planning,^19^ namely knowledge‐based planning (KBP), protocol‐based automatic iterative optimization (PB‐AIO), and multicriteria optimization (MCO). All of these paradigm have commercial implementation, such as Varian Rapid Plan(KBP),^20^ Pinnacle AutoPlan (PB‐AIO), RayStation (a posteriori MCO),^21^ and Erasmus‐iCycle (a priori lexicographic MCO). Our work followed closely to a previous work by Breedveld^7^, ^8^ on the Erasmus‐iCycle, a multicriterial and beam orientation optimization system. This system implemented a predefined wish‐list of plan evaluation criteria with a plan template for Monaco. Several studies^22^, ^23^ showed that Erasmus‐iCycle was able to produce better results than human planners in reduction of dose on critical structures. We realized that a lack of flexibility in template writing for auto‐planning with Monaco could potentially hinder its general clinical application and focused our effort in automation of template modification. The difference of optimization workflow between Erasmus‐iCycle and our platform is illustrated in Fig. 13. As seen in [Fig. 13(a)], the optimization process of planning template in Erasmus‐iCycle was separated from Monaco, and once the planning optimization finished, the patient‐specific plan template was generated from the plan and optimized into Monaco TPS. To avoid the difference between the external optimization and TPS inner optimization, all our plan optimization directly utilized the inner calculation and optimization engine in Monaco TPS, as shown in [Fig. 13(b)]. The template in our system served like a messenger between an external “virtual planner” and the TPS. The efficiency of optimal parameter search was helped with extracting the data from Monaco optimization process. The information extracted should reflect important geometric features of individual cases that have influences to the final results.

**FIG. 14:**
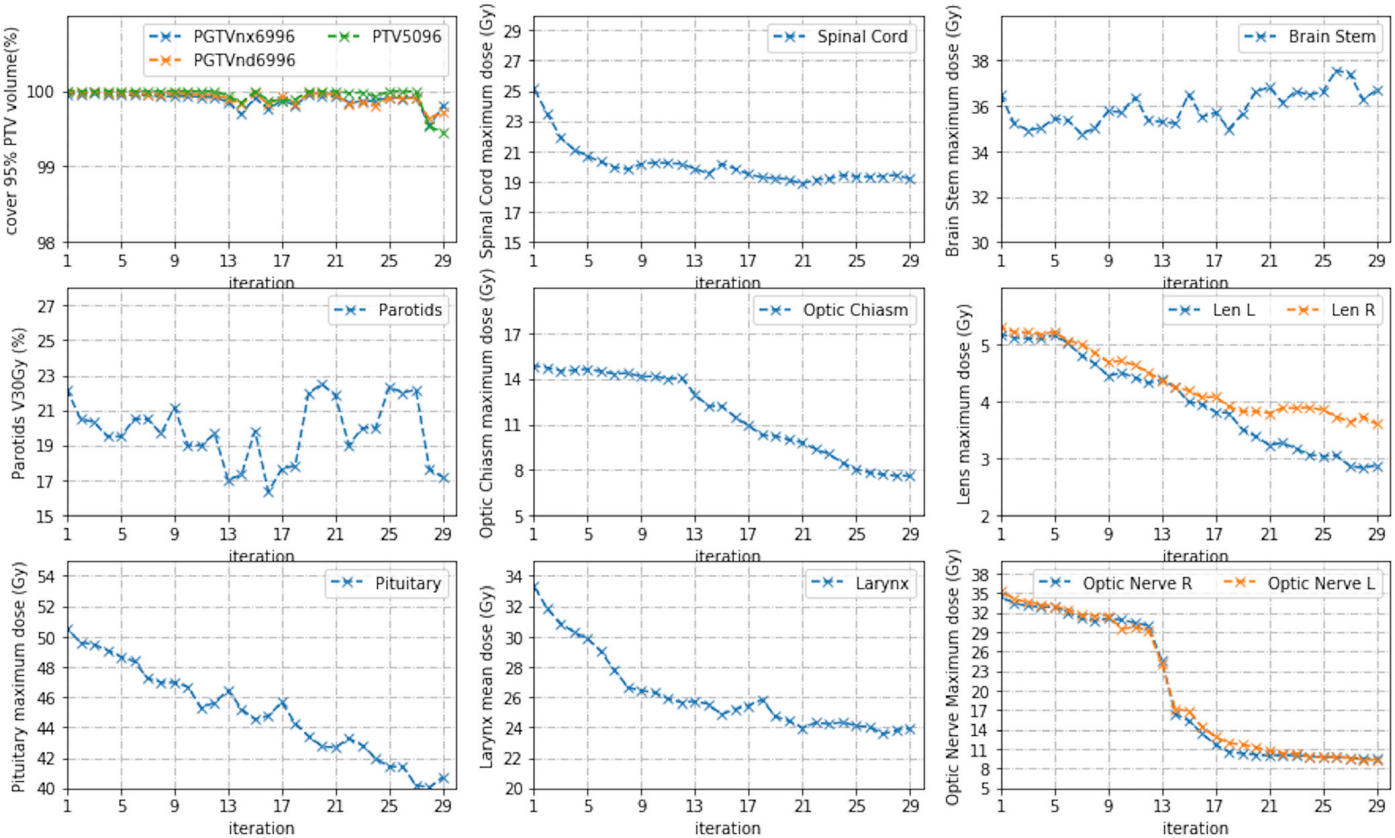
Change of dose–volume indices with iteration number for a head and neck case.

As seen in Fig. 14, for majority of our testing head and neck cases, most dose–volume indices converged to their objective values with no more than 30 iterations. However, as in any plan optimization, not all desired dosimetric goals could always be guaranteed. For example, the dose to parotid glands could converge in several iterations while the dose–volume metrics for the brain stem or spinal cord could not be consistently decreased but kept oscillating. When the optimization routine failed to meet a dosimetric constraint, two approaches could be attempted to mitigate the problem. One was to set the limit for the number of iterations, for example, 30–40 as in our implementation, in order to avoid the system being trapped in a dead loop. The performance of the initial template in terms of obtained overdose, relative impact, isoconstraints, and isoeffect from the iterative trials could be analyzed. Alternative template design could use a different beam configuration or a new set of constraint functions. The other was to set the priority of OARs with manually fixed constraint function weightings. For example, the constrains for the spinal cord or brain stem in head and neck cases could be set with an artificially high relative priority.

**FIG. 15:**
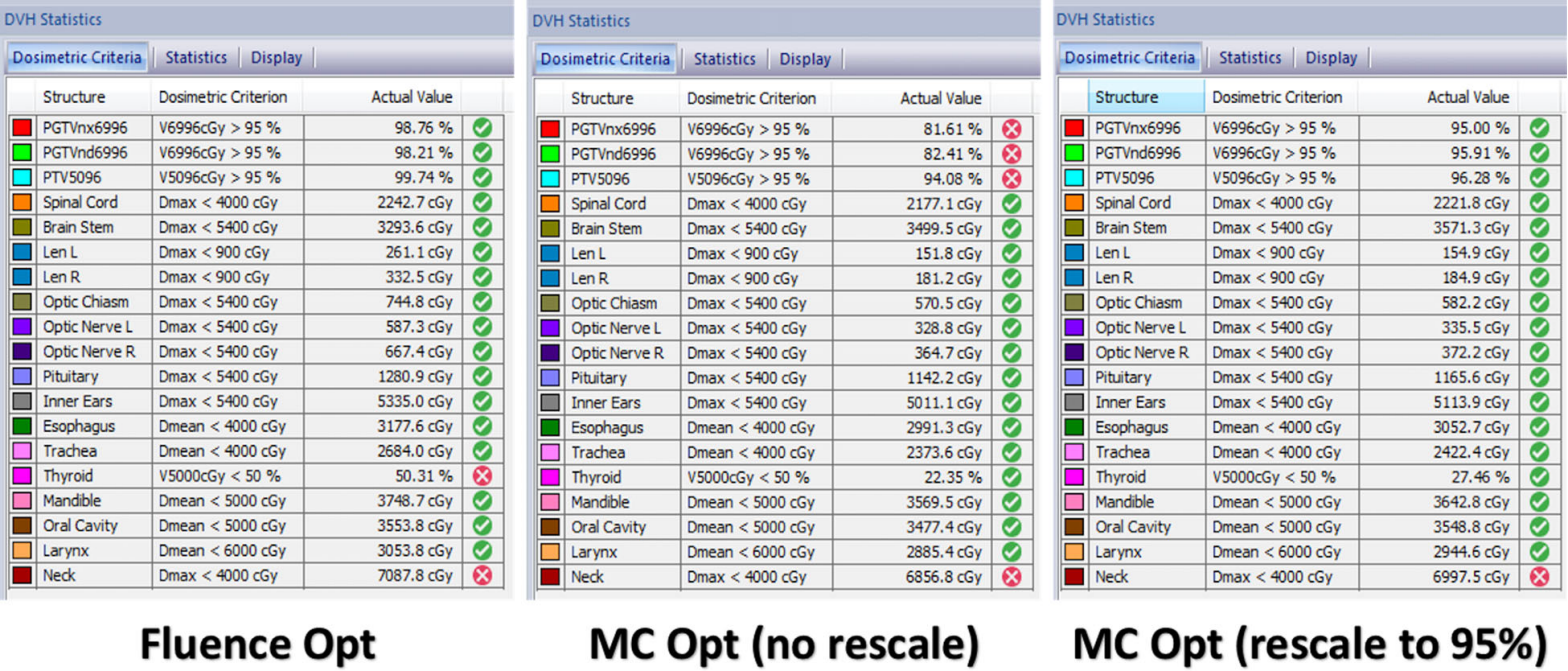
Comparison between FMO and segmentation optimization (without and with rescaling 95% dose coverage to PGTVnx6996). FMO, fluence map optimization.

**FIG. 16:**
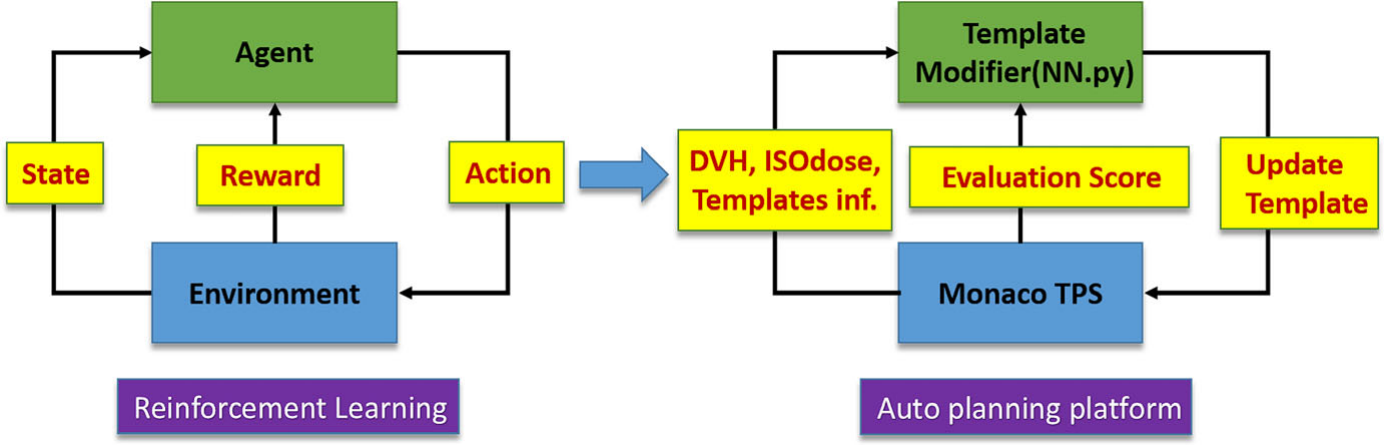
The similarity between deep reinforcement learning and auto treatment planning process.

The differences between FMO and the fully Monte Carlo calculated plan could be accepted by dose rescaling to meet the dose coverage requirement for the targets, as shown in Fig. 15. Zheng^24^ et al showed that the dose differences between FMO and the final optimization may be predictable. It is therefore possible to factor in an estimate for such differences in plan evaluation for Stage I optimization, as seen in Fig. 16.

Since it is difficult for a planner to track the planning process, a trial‐and‐error decision to change an optimization condition can be inefficient. Thus, reproducibility for the results is poor in general, especially among different planners. With auto‐planning, all the optimization parameters can be tracked and stored. After repeated planning trials are done, a planner can track each iteration history and evaluate multiple acceptable plans. Our parameters tuning method was different from popular neural network approach in that it does not require training data. We summarized the planning strategies into detailed steps and rules, and constructed all of them with python codes. Ten prostate and 10 head and neck cases were all used for validating the feasibility of our parameters tweaking method.

Currently, our auto‐planning platform was merged to Monaco 6.0 and with the help of inner scripting, the whole planning workflow was more efficient and robust. In future work, we plan to introduce reinforcement learning model, as illustrated by Fig. 16, to drive the whole IMRT parameters modification in each planning trial to avoid the underfitting problems. However, the action space may be tremendously large, and therefore, it needs an efficient and robust search method.

## 5 Conclusion

An auto‐planning platform to interface with Monaco was developed and tested with VMAT planning for prostate and for head and cases. Our preliminary work was focused on mimicking the interaction between an experienced planner and the TPS for specific treatment sites. This study showed that template‐based automation was feasible for clinical application showing acceptable results.

## Conflicts of Interest

No Conflicts of Interest.

## Supporting information


**Table S1**. Prescription of Prostate.
**Table S2**. Prescription of Head and neck case.
**Table S3**. DVH statistics comparison of 10 prostate cases.
**Table S4**. DVH statistics comparison of 10 head and neck cases.
**Table S5**. DVH statistics comparison between Stage I (FMO) and Stage II (segmentation optimization) in 10 prostate.
**Table S6**. DVH statistics comparison between Stage I (FMO) and Stage II 15 (segmentation optimization) in 10 head and neck.
