# Supplementary material for: A plan template‐based automation solution using a commercial treatment planning system
Source: J Appl Clin Med Phys. 2020 Mar 16;21(5):13–25. doi: 10.1002/acm2.12848 (PMC7286016; doi:10.1002/acm2.12848)
Supplement: Supplementary file 1 — Table S1. Prescription of Prostate. Table S2. Prescription of Head and neck case. Table S3. DVH statistics comparison of 10 prostate cases. Table S4. DVH statistics comparison of 10 head and neck cases. Table S5. DVH statistics comparison between Stage I (FMO) and Stage II (segmentation optimization) in 10 prostate. Table S6. DVH statistics comparison between Stage I (FMO) and Stage II 15 (segmentation optimization) in 10 head and neck. [file ACM2-21-13-s001.pdf]

Supplementary Material

Table 1. Prescription of Prostate case.

Organ Indices	Evaluation Criteria	Prescription Requests
PTV6750	Minimum Volume of PTV6750 covered by 67.5Gy	95.0(%)
PTV4750	Minimum Volume of PTV4750 covered by 47.5Gy	95.0(%)
Rectum1	Maximum Volume of Rectum covered by 40.0Gy	30.0(%)
Rectum2	Maximum Volume of Rectum covered by 50.0Gy	20.0(%)
Rectum3	Maximum Volume of Rectum covered by 62.5Gy	5.0(%)
Bladder1	Maximum Volume of Rectum covered by 40.0Gy	30.0(%)
Bladder2	Maximum Volume of Rectum covered by 50.0Gy	20.0(%)
Bladder3	Maximum Volume of Rectum covered by 60.0Gy	10.0(%)
Femoral Head	Maximum Dose	54(Gy)

Table 2. Prescription of Head and neck case.

Organ Indices	Evaluation Criteria	Prescription Requests
PTVnx6996	Minimum Volume of PTVnx6996 covered by 69.96Gy	95.0(%)
PTVnd6996	Minimum Volume of PTVnd6996 covered by 69.96Gy	95.0(%)
PTV5096	Minimum Volume of PTV5096 covered by 50.96Gy	95.0(%)
Spinal Cord	Maximum Dose	40.0(Gy)
Cord PRV	Maximum Dose	45.0(Gy)
Brain Stem	Maximum Dose	54.0(Gy)
Stem PRV	Maximum Dose	60.0(Gy)
Len L	Maximum Dose	9.0(Gy)
Len R	Maximum Dose	9.0(Gy)
Optical Chiasm	Maximum Dose	54.0(Gy)
Optical Nerve L	Maximum Dose	54.0(Gy)
Optical Nerve R	Maximum Dose	54.0(Gy)
Pituitary	Maximum Dose	54.0(Gy)
TM L	Maximum Dose	54.0(Gy)
TM R	Maximum Dose	54.0(Gy)
Inner Ears	Maximum Dose	54.0(Gy)
Parotids	Maximum Volume of Parotids covered by 50Gy	30.0(%)
Esophagus	Maximum Mean Dose	40.0(Gy)
Trachea	Maximum Mean Dose	40.0(Gy)
Thyroid	Maximum Volume of Thyroid covered by 50Gy	50.0(%)
Mandible	Maximum Mean Dose	50.0(Gy)
Oral Cavity	Maximum Mean Dose	50.0(Gy)
Larynx	Maximum Mean Dose	60.0(Gy)
Neck	Maximum Dose	40.0(Gy)

Table 3. DVH statistics comparison of 10 prostate cases.

Prescription	Clinical(mean±std)	Auto(mean±std)	P value
PTV6750 (V67.5Gy ≥ 95%)	96.35±1.76	95.12±0.21	0.063
PTV4750 (V47.5Gy ≥ 95%)	96.14±1.37	96.55±0.75	0.285
Bladder1 (V60Gy ≤ 10%)	6.57±6.60	5.37±3.88	0.721
Bladder2 (V50Gy ≤ 20%)	10.65±9.40	10.82±8.51	0.878
Bladder3 (V40Gy ≤ 30%)	24.74±9.53	22.65±8.61	0.114
Rectum1 (V62.5Gy ≤ 5%)	2.83±2.15	1.63±0.97	0.059
Rectum2 (V50Gy ≤ 20%)	12.79±3.65	9.58±4.20	0.092
Rectum3 (V40Gy ≤ 30%)	26.06±5.96	21.42±6.40	0.139
Pubic Bone (V62.5Gy ≤ 15%)	6.39±4.61	3.92±3.58	0.086
Femoral Head L(Dmax ≤ 54Gy)	41.63±2.91	40.62±2.57	0.646
Femoral Head R(Dmax ≤ 54Gy)	40.70±2.73	40.80±3.26	0.799
Maximum Dose(Dmax ≤ 74.25Gy)	73.00±1.10	73.46±1.14	0.203

Table 4. DVH statistics comparison of 10 head and neck cases.

Prescription	Clinical(mean±std)	Auto(mean±std)	P value
PTVnx6996 (V69.96Gy ≥ 95%)	96.92±1.21	96.02±1.57	0.341
PTVnd6996 (V69.96y ≥ 95%)	98.02±1.30	97.01±1.80	0.084
PTV5096(V50.96Gy ≥ 95%)	97.53±1.79	96.26±1.49	0.097
Brain Stem (Dmax ≤ 54Gy)	45.85±4.47	42.23±5.09	0.160
Spinal Cord (Dmax ≤ 40Gy)	33.63±1.53	28.48±4.87	0.027
Left Len (Dmax ≤ 9Gy)	3.05±1.98	3.78±2.29	0.027
Right Len (Dmax ≤ 9Gy)	3.23±1.72	3.73±1.98	0.160
Left Optic Nerve (Dmax ≤ 54Gy)	18.96±17.10	19.28±18.09	0.846
Right Optic Nerve (Dmax ≤ 54Gy)	23.11±17.27	19.36±18.27	0.492
Optic Chiasm (Dmax ≤ 54Gy)	21.50±16.88	22.77±18.68	0.625
Parotids (V50Gy ≤ 30%)	29.03±10.98	26.96±8.58	0.004
Esophagus (Dmean ≤ 40Gy)	29.74±5.19	31.21±6.03	0.322
Mandible (Dmean ≤ 50Gy)	38.54±4.68	34.98±4.19	0.010
Maximum (Dose ≤ 76.95Gy)	76.45±0.62	77.89±1.24	0.027

Table 5. DVH statistics comparison between Stage I (FMO) and Stage II (segmentation optimization) in 10 prostate cases.

Prescription	Stage I(mean±std)	Stage II(mean±std)	P value
PTV6750 (V67.5Gy ≥ 95%)	97.52±1.65	95.12±0.21	0.033
PTV4750 (V47.5Gy ≥ 95%)	99.13±0.65	96.55±0.75	0.008
Bladder1 (V60Gy ≤ 10%)	4.71±3.52	5.37±3.88	0.260
Bladder2 (V50Gy ≤ 20%)	10.45±8.68	10.82±8.51	0.441
Bladder3 (V40Gy ≤ 30%)	21.86±9.78	22.65±8.61	0.110

Rectum1 (V62.5Gy ≤ 5%)	1.52±1.12	1.63±0.97	0.953
Rectum2 (V50Gy ≤ 20%)	7.61±3.62	9.58±4.20	0.015
Rectum3 (V40Gy ≤ 30%)	18.23±5.71	21.42±6.40	0.038
Pubic Bone (V62.5Gy ≤ 15%)	3.40±3.38	3.92±3.58	0.311
Femoral Head L (Dmax ≤ 54Gy)	40.31±1.97	40.62±2.57	0.953
Femoral Head R (Dmax ≤ 54Gy)	41.02±2.34	40.80±3.26	0.767
Maximum (Dmax ≤ 74.25Gy)	71.49±1.08	73.46±1.14	0.011

Table 6. DVH statistics comparison between Stage I (FMO) and Stage II (segmentation optimization) in 10 head and neck cases.

**Prescription**	**Stage I(mean±std)**	**Stage II(mean±std)**	**P value**
PTVnx6996 (V69.96Gy ≥ 95%)	98.51±1.56	96.02±1.57	0.009
PTVnd6996 (V69.96y ≥ 95%)	98.41±1.33	97.01±1.80	0.075
PTV5096(V50.96Gy ≥ 95%)	99.51±0.71	96.26±1.49	0.005
Brain Stem (Dmax ≤ 54Gy)	39.34±5.73	42.23±5.09	0.013
Spinal Cord (Dmax ≤ 40Gy)	26.83±4.14	28.48±4.87	0.047
Left Len (Dmax ≤ 9Gy)	4.70±1.83	3.78±2.29	0.013
Right Len (Dmax ≤ 9Gy)	5.01±1.89	3.73±1.98	0.009
Left Optic Nerve (Dmax ≤ 54Gy)	23.16±16.47	19.28±18.09	0.093
Right Optic Nerve (Dmax ≤ 54Gy)	23.11±17.27	19.36±18.27	0.139
Optic Chiasm (Dmax ≤ 54Gy)	22.42±16.65	22.77±18.68	0.059
Parotids (V50Gy ≤ 30%)	21.03±9.00	26.96±8.58	0.005
Esophagus (Dmean ≤ 40Gy)	31.90±5.72	31.21±6.03	0.022
Mandible (Dmean ≤ 50Gy)	36.93±3.73	34.98±4.19	0.005
Maximum (Dose ≤ 76.95Gy)	74.56±1.13	77.89±1.24	0.005
